# Multipoint pacing is associated with reduction of heart failure hospitalizations or death in patients who do not respond to cardiac resynchronization therapy: results of the MORE-CRT MPP randomized trial

**DOI:** 10.1093/europace/euaf070

**Published:** 2025-03-30

**Authors:** Christophe Leclercq, Haran Burri, Leonardo Calò, Christopher Aldo Rinaldi, Johannes Sperzel, Bernard Thibault, Tim Betts, Pascal Defaye, Andreas Hain, Olivier Piot, Kwangdeok Lee, Wenjiao Lin, Annalisa Pollastrelli, Andrea Grammatico, Giuseppe Boriani

**Affiliations:** Service de Cardiologie et Maladies Vasculaires, rue Henri le Guilloux 35033 Rennes Cédex 09, CHRU Hopital de Pontchaillou, Rennes, France; Département de Cardiologie, Hôpital Cantonal Universitaire de Geneva, Geneva, Switzerland; Department of Cardiology, Policlinico Casilino, Rome, Italy; Department of Cardiology, St. Thomas’ Hospital, London, UK; Department of Cardiology, Kerckhoff Heart and Thorax Center, Bad Nauheim, Germany; Département de Cardiologie, Institut de Cardiologie de Montreal, Montreal, Canada; Department of Cardiology, John Radcliffe Hospital, Oxford, UK; Département de Cardiologie, Grenoble Alpes University Hospital and Grenoble Alpes University, Grenoble, France; Department of Cardiology, Kerckhoff Heart and Thorax Center, Bad Nauheim, Germany; Service de Cardiologie, Centre Cardiologique Du Nord, Saint-Denis, France; Abbott Clinical, Plano, USA; Abbott Clinical, Plano, USA; Abbott Medical Affairs, Rome, Italy; Abbott Medical Affairs, Rome, Italy; Department of Cardiology, Università degli Studi di Modena e Reggio Emilia, Modena Italy

**Keywords:** Cardiac resynchronization therapy, Multipoint pacing, Heart failure hospitalizations, All-cause mortality

## Abstract

**Aims:**

Cardiac resynchronization therapy (CRT) via biventricular pacing (BIVP) is an effective treatment, but non-responders are at a higher risk of death and heart failure (HF) hospitalizations compared with CRT responders. The MORE-CRT MPP trial aimed to evaluate whether CRT with multipoint pacing (MPP) is associated with improved clinical outcomes in CRT non-responders.

**Methods and results:**

Cardiac resynchronization therapy patients were treated with conventional BIVP for 6 months and then assessed for CRT response (left ventricular end-systolic volume relative reduction >15% vs. baseline). Cardiac resynchronization therapy non-responders were 1:1 randomized to BIVP or MPP and followed for 6 months. The main endpoint of this secondary analysis was HF hospitalizations or all-cause mortality. Of 3724 CRT patients (67 ± 11 years, 1050 female), 1677 were non-responders and randomized to MPP or BIVP, of whom 1421 (722 MPP and 699 BIVP) had complete data. In a mean follow-up of 5 ± 1 months after randomization, MPP was associated with a lower incidence of HF hospitalizations or all-cause mortality [48/722 (6.64%)] compared with BIVP (73/699 (10.44%), RRR = 36% (95% CI=±4%), *P* = 0.0107). At multivariable analysis, MPP was associated with a lower occurrence of the main endpoint (odds ratio = 0.60, *P* = 0.0124). At logistic regression analysis, HF hospitalizations or all-cause death were lower with MPP vs. BIVP in the whole population and in many patients subgroups, e.g. ischaemic patients and patients with long (>105 ms) interventricular electrical delay.

**Conclusion:**

In the MORE-CRT MPP randomized trial, MPP was associated with a significant reduction of all-cause mortality and HF hospitalizations in prior non-responders to conventional biventricular pacing.

## Graphical Abstract

**Graphical Abstract:**
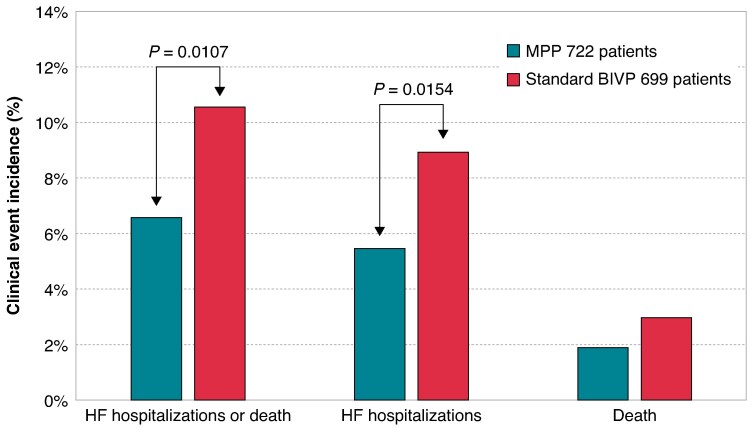

**What’s new**

- The MORE-CRT MPP trial is the largest randomized study designed to assess cardiac resynchronization therapy response in patients in whom conventional biventricular pacing (BIVP) does not induce a clinically relevant left ventricle reverse remodelling.
- The results described in this article show that multipoint pacing (MPP), in patients who do not respond to BIVP, is associated with clinically relevant and statistically significant reduction of hard endpoints, such as heart failure hospitalizations or all-cause mortality.
- A significantly better outcome was observed with MPP, compared with BIVP, in the whole population and in specific patient subgroups, such as ischaemic patients, patients with very wide (≥160 ms) QRS, and patients with long (>105 ms) interventricular electrical delay, suggesting that MPP, by delivering sequential pacing from two left ventricular pacing sites, captures large areas of the left ventricle and may benefit patients with scars and patients with large interventricular electrical delay.

## Introduction

Cardiac resynchronization therapy (CRT) via biventricular pacing (BIVP) is an established treatment for selected patients with heart failure (HF), left ventricular (LV) systolic dysfunction, reduced LV ejection fraction and wide QRS.^1–3^

Multiple prospective randomized trials have accumulated strong evidence of CRT benefits in terms of survival, reduction in HF hospitalizations, LV reverse remodelling, exercise capacity and quality of life.^4–9^ Nevertheless, still today, a significant fraction of patients does not respond to CRT, possibly due to non-optimal patient selection, and/or sub-optimal LV lead placement or device programming, or other conditions.^10–12^

Technological advances in CRT tools, such as quadripolar leads^13^ and MultiPoint™ Pacing (MPP)^14,15^ have been proposed to reach the best LV pacing site and to deliver optimal sequential pacing from two LV pacing sites, with the aim to overcome unfavourable coronary venous anatomies, presence of ischaemic scar, phrenic nerve stimulation, high capture thresholds, and to provide improved CRT.

Four randomized studies^16–19^ have so far compared MPP and BIVP. Three studies evaluated the endpoint of LV reverse remodelling applying MPP with three different strategies: as a first-line strategy,^16^ as an added strategy in patients who responded to CRT,^17^ and as an added strategy in patients who did not respond to CRT.^18^ A significant improvement of LV reverse remodelling with MPP was reported by AlMusaad et al.^16^ and by Marques et al.^17^ but not in the Cardiac Resynchronization Therapy with MultiPoint Pacing (MORE-CRT MPP) trial.^18^ The fourth study did not find statistical differences between MPP and BIVP in the endpoint composed by all-cause mortality, HF-related hospitalization, New York Heart Association functional class, and the patient global assessment score.^19^

Cardiac resynchronization therapy non-responders were identified as subjects with a higher risk of death and HF hospitalizations, as well as higher healthcare expenditures, compared with CRT responders.^12,20,21^

We have performed a secondary analysis of the MORE-CRT MPP trial data to evaluate, whether MPP is associated with reduced incidence of HF hospitalizations or death from any cause, compared with BIVP, in patients who do not respond to CRT.

## Methods

The MORE-CRT MPP trial was a prospective, randomized, international multi-centre study (ClinicalTrials.gov Identifier: NCT02006069) that enrolled patients at 215 centres worldwide.^18^

The study was approved by the Ethics Committee of all participating centres and was conducted in compliance with the Declaration of Helsinki. All patients provided written informed consent.

The study was designed with a 6-month long observational phase in which all patients were programmed with BIVP. At their 6 month follow-up, patients without reverse remodelling [i.e. patients with LV end-systolic volume (LVESV) relative reduction <15% comparing baseline and 6 months measurements] were 1:1 randomized in two arms, one continuing with BIVP and one adding the MPP algorithm.^18^

Clinical outcomes were assessed in the 6 months after randomization.

### Study participants

The MORE-CRT MPP study enrolled patients with a CRT indication according to current International Guidelines at the time of the trial.^22^ The complete list of inclusion and exclusion criteria is reported in the Supplementary Data.

### Cardiac resynchronization therapy device and MPP algorithm

Commercially available cardiac resynchronization therapy devices (Quadra, Abbott, Sylmar, CA) and quadripolar LV leads (Quartet^TM^ LV lead, Abbott, Sylmar, CA) were implanted in this study. Implantation was performed according to the clinical practice of the individual centres. The LV lead location was classified by investigators using fluoroscopic imaging, according to standard criteria, as apical or non-apical (basal or midventricular) in the right anterior oblique projection, as well as anterior, posterior, or lateral in the left anterior oblique projection.

These devices feature the MPP algorithm, which allows sequential pacing pulses to be delivered from two sites on the same LV lead, potentially capturing a larger area and engaging multiple zones in the long axis of the LV.

### Device programming

At implant and during the following 6 months, CRT devices were programmed to deliver BIVP with the LV pacing vector and AV delay settings at the physician’s discretion.

### Interventricular electrical delays

Some authors have suggested that interventricular electrical delay, measured as the time difference between right ventricle (RV) lead and LV lead sensing, is associated with CRT response.^23–25^ In our study interventricular electrical delays were measured for each patient, as the time difference between sensing at the RV lead and sensing at each of the four electrodes of the quadripolar LV lead (D1, M2, M3, and P4) during intrinsic conduction. Each patient was therefore characterized by a mean interventricular electrical delay (sRV-sLV), estimated as the average of the four interventricular electrical delays measured at 6-month visit when randomization occurred.

### Analyses objective

The objective of the analysis was to evaluate whether MPP is associated with reduced incidence of HF hospitalizations or all-cause mortality, compared with BIVP, in patients who do not respond to CRT. HF hospitalizations and death for any cause were pre-specified secondary endpoints of the study.

### Statistical analysis

For the analyses described in this manuscript, we selected the cohort of patients who completed the first observational phase of the study, had complete intrinsic interventricular electrical delay data, had complete echocardiographic data at baseline and at 6 months after implant, were classified as CRT non-responders, and were randomized between MPP and BIVP.

Data of continuous variables that follow a normal distribution are presented as mean and standard deviation, while data of continuous variables that follow a skewed distribution are presented as median and interquartile range. Comparisons of continuous variables having a normal distribution were performed with the Student’s *t*-test. Discrete variables are presented as numbers and proportions. Proportions were compared with Fisher’s exact or Pearson chi-square tests, as appropriate.

Incidence of clinical outcomes, such as HF hospitalizations, any-cause death and the endpoint composed by HF hospitalizations or any-cause death, was evaluated either as the raw incidence, i.e. the percentage of patients with events, or as Kaplan–Meier freedom. Composite endpoint raw incidences in the two randomized patient groups were compared with Chi-Square proportion test, while Kaplan–Meier freedom from composite endpoint in the two randomized patient groups was compared with the Log Rank test.

Composite endpoint relative risk reduction (RRR), comparing the two randomized patient groups, was expressed in percentage with its lower and upper 95% confidence interval (95% CI).

Univariable and multivariable logistic regression models were used to analyze the association between the risk of the endpoint composed by HF hospitalizations or any-cause death and patient characteristics and device programming at randomization.

Forest plot analyses were used to determine which patient subgroups had better clinical outcomes with MPP compared with BIVP.

SAS version 9.4 (SAS Institute, Cary, North Carolina) was used for statistical analysis.

### Results

**Figure 1:**
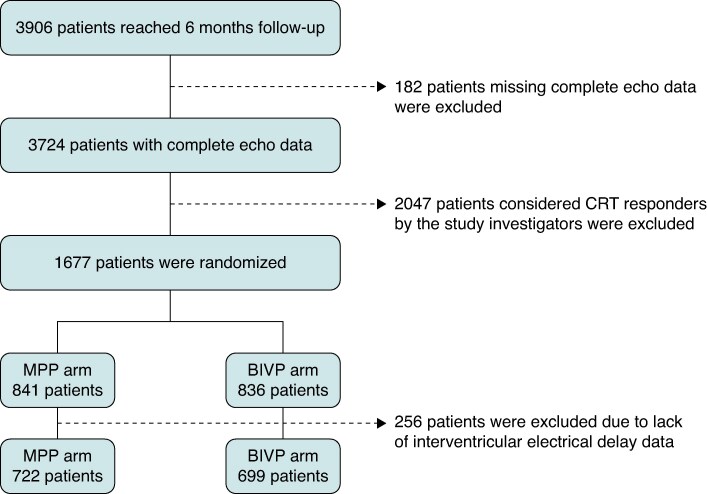
Study flow chart.

As shown in *Figure 1*, 3906 patients were initially followed up for 6 months. Complete echocardiographic data were available for the sub-group of 3724 patients, 1766 were classified as CRT non-responders by the study investigators and were randomly programmed to MPP or BIVP. For these analyses, we selected the sub-group of 1421 patients who had complete data about the interventricular electrical delay at baseline and at 6 months, who, according to the protocol, had been randomized to MPP (722 patients) or BIVP (699 patients).

Patients’ characteristics at randomization are shown in *Table 1*. Table *A* in the Supplementary Data shows that the patient characteristics were not statistically different when comparing the two randomized groups. Table *B* in the Supplementary Data shows the pharmacological treatment of the whole population and the two randomized groups.

**Table 1:** Patient characteristics

	Subjects analyzed (*n* = 1421)	MPP ON (*n* = 722)	MPP OFF (*n* = 699)
Age in years, mean ± st dev (*n*)	68 ± 10 (1421)	68 ± 10 (722)	68 ± 11 (699)
Female gender, % (*n/N*)	23.1 (328/1421)	21.7 (157/722)	24.5 (171/699)
NYHA Class II, % (*n/N*)	49.0 (697/1421)	50.1 (362/722)	47.9 (335/699)
NYHA Class III, % (*n/N*)	48.5 (689/1421)	47.2 (341/722)	49.8 (348/699)
NYHA Class IV, % (*n/N*)	2.2 (31/1421)	2.4 (17/722)	2.0 (14/699)
Ischaemic cardiomyopathy, % (*n/N*)	51.0 (725/1421)	52.5 (379/722)	49.5 (346/699)
Hypertension, % (*n/N*)	61.7 (877/1421)	61.9 (447/722)	61.5 (430/699)
Hypercholesterolemia, % (*n/N*)	42.7 (607/1421)	45.2 (326/722)	40.2 (281/699)
Diabetes mellitus, % (*n/N*)	37.7 (536/1421)	36.8 (266/722)	38.6 (270/699)
COPD, % (*n/N*)	10.7 (152/1421)	10.5 (76/722)	10.9 (76/699)
Renal disease, % (*n/N*)	19.4 (276/1421)	19.9 (144/722)	18.9 (132/699)
Active smoker, % (*n/N*)	9.7 (138/1421)	9.7 (70/722)	9.7 (68/699)
Ex-smoker, % (*n/N*)	35.3 (501/1421)	34.2 (247/722)	36.3 (254/699)
Stroke, % (*n/N*)	4.2 (59/1421)	5.0 (36/722)	3.3 (23/699)
Transient ischaemic attack, % (*n/N*)	3.2 (46/1421)	4.2 (30/722)	2.3 (16/699)
Peripheral artery disease, % (*n/N*)	5.8 (83/1421)	5.4 (39/722)	6.3 (44/699)
Neoplastic disease, % (*n/N*)	4.0 (57/1421)	4.0 (29/722)	4.0 (28/699)
Thyroid dysfunction, % (*n/N*)	9.5 (135/1421)	9.1 (66/722)	9.9 (69/699)
LBBB, % (*n/N*)	63.2 (781/1236)	62.8 (391/623)	63.6 (390/613)
RBBB, % (*n/N*)	7.7 (95/1236)	8.8 (55/623)	6.5 (40/613)
Left anterior fascicular block, % (*n/N*)	6.7 (83/1236)	6.3 (39/623)	7.2 (44/613)
Left posterior fascicular block, % (*n/N*)	0.2 (2/1236)	0.3 (2/623)	0.0 (0/613)
Intraventricular conduction delay, % (*n/N*)	24.1 (298/1236)	23.8 (148/623)	24.5 (150/613)
QRS duration in ms, mean ± st dev (*N*)	155 ± 25 (1421)	155 ± 26 (722)	155 ± 24 (699)
LVESV in ml, mean ± st dev (*N*)	155 ± 66 (1389)	155 ± 65 (710)	155 ± 67 (679)
LVEDV in ml, mean ± st dev (*N*)	216 ± 77 (1389)	216 ± 77 (710)	215 ± 78 (679)
LVEF %, mean ± st dev (*N*)	29 ± 8 (1389)	29 ± 8 (710)	29 ± 8 (679)
CRT-D, % (*n/N*)	91.8 (1305/1421)	91.7 (662/722)	92.0 (643/699)

No statistical differences between the two groups were observed for all parameters.

NYHA, New York Heart Association; COPD, Chronic Obstructive Pulmonary Disease; CRT-D, cardiac resynchronization defibrillator; LVESV, left ventricle end-systolic volume; LVEF, left ventricle ejection fraction; LBBB, left bundle branch block; RBBB, right bundle branch block

The RV lead was placed in the RV apex in 62.2% of patients, in RV septum in 33.8% of patients and in other RV sites for 4% of patients. The LV leads were positioned through the coronary sinus on the lateral LV wall in 42.3% of patients, on the posterior lateral LV wall in 40.6% of patients, on the anterior lateral wall in 9.9% of patients and other locations in 7.2% of patients. The LV lead position was midventricular in 69.2% of patients and basal in 22.1% of patients. Lead positions were not statistically different when comparing the two randomized groups.

At 6-month visit, when randomization occurred, the median (interquartile range) percentage of BIVP, delivered after implant, was 98% (93–100%), the median (interquartile range) of sRV-sLV was 105 ms (80–135 ms), the average of sensed AV delay was 110 ± 10 ms and the average of paced AV delay was 144 ± 12 ms; the distributions of these variables were not statistically different when comparing the two randomized groups.

## Clinical outcomes

**Figure 2:**
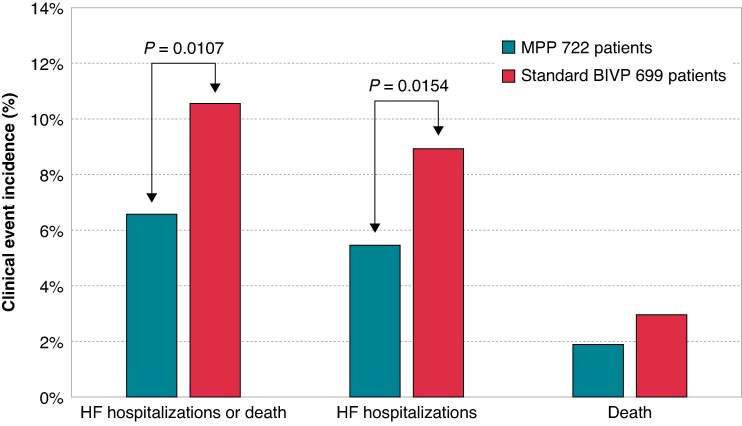
Raw incidences of clinical events in 1421 randomized patients.

In a mean follow-up of 5 ± 1 months after randomization, HF hospitalizations or any-cause death occurred in 121/1421 (8.5%) patients. As shown in *Figure 2*, MPP was associated with a lower incidence of HF hospitalizations or all-cause mortality [48/722 (6.64%)] compared with BIVP (73/699 (10.44%), RRR = 36% (95% CI=±4%), *P* = 0.0107). In particular, in LBBB patients, the composite endpoint incidence was 3.72% in the MPP arm and 7.2% [RRR = 48% (95% CI=±4%), *P* = 0.0480] in BIVP arm and, in non-LBBB patients, it was 8.33% in the MPP arm and 15.84% [RRR = 47% (95% CI=±4%), *P* = 0.0228] in BIVP arm.

HF hospitalizations occurred in 101/1421 (7.1%) patients. Multipoint pacing was associated with a lower incidence of HF hospitalizations [39/722 (5.4%)] compared with BIVP (62/699 (8.9%), RRR = 39% (95% CI=±4%), *P* = 0.0154). Death occurred in 34/1421 (2.4%) patients, specifically 14/722 (1.9%) patients in the MVP arm and in 20/699 (2.9%) patients in the BIVP arm (*P* > 0.05). Multipoint pacing was associated with a lower incidence of HF hospitalizations or all-cause mortality also in all 1677 randomized patients as shown in *Figure A* in the Supplementary Data.

**Figure 3:**
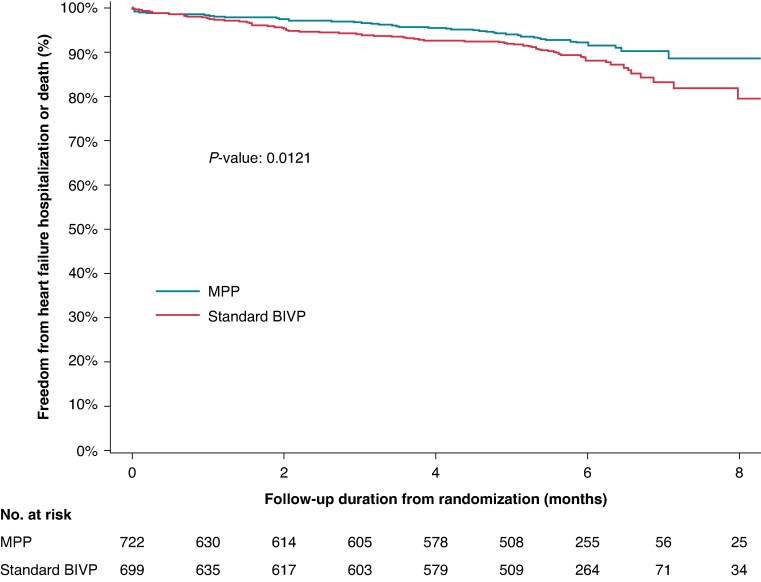
Kaplan–Meier freedom from HF hospitalizations or all-cause death.

As shown in *Figure 3*, at Kaplan–Meier analysis, the incidence of HF hospitalizations or death was significantly lower in Multipoint pacing patients compared with BIVP patients. In particular, at 8 months after randomization, it was 11.5% in MPP patients and 18.2% in BIVP patients, RRR = 37% (95% CI=±4%) *P* = 0.0121.

Univariable and multivariable logistic analyses confirmed that patients randomly assigned to MPP had a lower risk of the composite endpoint of HF hospitalizations or all-cause mortality, as shown in *Table 2*. In particular, at multivariable analysis, MPP programming was associated with a 40% risk reduction of the composite endpoint [odds ratio = 0.60 (95% CI = 0.40–0.89), *P* = 0.0124] compared with BIVP. Moreover, at multivariable analysis, LBBB and high LVEF were associated with a risk reduction of the composite endpoint, while the renal disease was associated with a risk increase.

**Table 2:** Risk of HF hospitalizations or any-cause death at univariable and multivariable analysis

	Univariable analysis		Multivariable analysis	
Parameters	Odds ratio [95% CI]	*P* value	Odds ratio [95% CI]	*P* value
AF yes vs. no	0.575 [0.076, 4.354]	0.5925		
Age	0.998 [0.981, 1.015]	0.8161		
COPD yes vs. no	0.656 [0.337, 1.279]	0.2161		
Diabetes yes vs. no	1.402 [0.979, 2.009]	0.0655		
Hypercholesterolemia yes vs. no	0.819 [0.568, 1.181]	0.2851		
Hypertension yes vs. no	1.109 [0.765, 1.608]	0.5849		
Ischaemic vs. non-ischaemic	0.972 [0.680, 1.389]	0.8759		
LBBB vs. non-LBBB	0.622 [0.419, 0.922]	0.0180	0.61 [0.40, 0.90]	0.0144
LVEF	0.966 [0.942, 0.991]	0.0080	0.96 [0.93, 0.99]	0.0056
LVESV	1.003 [1.001, 1.006]	0.0045		
LV Lead Apical vs. non-Apical	0.938 [0.491, 1.790]	0.8450		
NYHA Class II vs. III/IV	0.645 [0.447, 0.930]	0.0188		
QRS	1.002 [0.994, 1.010]	0.6322		
Renal Disease Yes vs. No	2.079 [1.405, 3.077]	0.0003	2.17 [1.40, 3.36]	0.0006
Female vs. Male	1.161 [0.770, 1.751]	0.4759		
MPP vs. BIVP randomization	0.572 [0.397, 0.825]	0.0028	0.59 [0.39, 0.89]	0.0116
BIVP% > 97% vs. ≤ 97%	0.720 [0.498, 1.042]	0.0817		
sRV-sLV > 105 ms vs. ≤ 105 ms	0.789 [0.549, 1.134]	0.2000		

AF, atrial tachyarrhythmias history; COPD, Chronic Obstructive Pulmonary Disease; LBBB, left bundle branch block; LVEF, left ventricle ejection fraction; LVESV, left ventricle end-systolic volume; LV, left ventricle; NYHA, New York Heart Association; MPP, multipoint pacing; BIVP%, biventricular pacing percentage during the first 6 months (before randomization); sRV-sLV, interventricular electrical delay as measured at 6 months visit (randomization).

**Figure 4:**
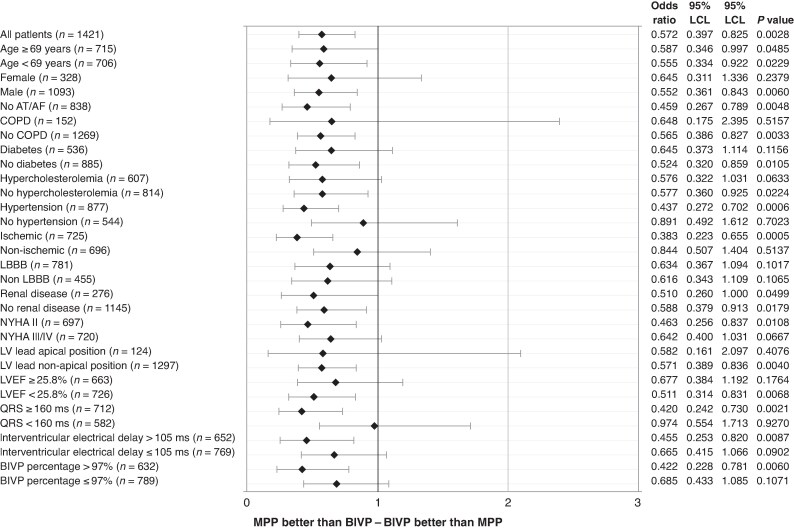
Risk of HF hospitalization or any-cause death between MPP and BIVP.

Logistic regression analysis showed that the risk of HF hospitalizations or any-cause death was reduced by MPP vs. BIVP by 42.8% (odds ratio = 0.572, *P* = 0.0028) in the whole population and several patient subgroups, as shown in *Figure 4*. In particular, HF hospitalizations or all-cause mortality risk was reduced by MPP vs. BIVP by 61.7% (*P* = 0.0005) in ischaemic patients, by 58% (*P* = 0.0021) in patients with very wide (≥160 ms) QRS, by 57.8% (*P* = 0.0060) in patients with a high (>97%) BIVP percentage, by 56.3% (*P* = 0.0006) in patients with hypertension, by 54.5% (*P* = 0.0087) in patients with long (>105 ms) interventricular electrical delay, by 54.1% (*P* = 0.0048) in patients without atrial tachyarrhythmias.

## Discussion

Our secondary analyses of the MORE-CRT MPP randomized trial show that MPP is associated with a clinically relevant and statistically significant reduction of HF hospitalizations or death in patients who do not respond to BIVP. Better clinical outcomes with MPP were observed in the whole population and in several patient subgroups, for example in patients with long interventricular electrical delay and in patients with ischaemic cardiomyopathy.

### Multipoint pacing compared with BIVP

Multipoint pacing, which allows sequential pacing pulses to be delivered from two LV sites rather than from one site only, as in standard BIVP, was developed to address some of the conditions which may cause CRT non-response. Several observational acute studies^14,15,26,27^ and two small, randomized studies^16,17^ have associated MPP with faster depolarization velocity, shorter LV conduction times, improved LV intraventricular dyssynchrony, increased cardiac output and improved LV reverse remodelling. However, the large MORE-CRT MPP randomized study^18^ did not show a significant difference between MPP and BIVP, in terms of CRT non-responders, when echocardiographic LV reverse remodelling (LVESV relative reduction >15%) was evaluated. Also the MPP trial did not show significant differences comparing MPP and BIVP in terms of a clinical composite score.^19^ We now report additional analyses of the MORE-CRT MPP trial utilizing a clinical endpoint—freedom from HF hospitalizations or all-cause mortality—which is of great importance for clinical practice, It is important to outline the fact that MPP was associated with a better clinical outcome in a relatively short period (6 months), making the observed difference (3.8% absolute reduction and 36% RRR of HF hospitalizations or any-cause death raw incidence) not only statistically significant but also clinically relevant, especially considering that the evaluated population initially did not respond to CRT. These findings derive from a large randomized controlled study, using an intention-to-treat approach and multivariable models to discard possible confounders. These new results re-enforce findings of both randomized^16,17^ and observational^14,15,26,27^ previous studies. An intriguing question is to understand why we observed these positive results despite the fact that MPP previously was not associated with improved clinical composite score vs. BIVP, in the MPP trial,^19^ and was not associated with better LV reverse remodelling vs. BIVP, in previous analyses of the MORE-CRT MPP trial.^18^ As for the comparison with the MPP trial^19^ we believe that different results of MORE-CRT MPP trial may derive from important methodological differences between the two studies such as (1) a different sample size (1677 patients were randomized in the MORE-CRT MPP trial compared with 381 patients in the MPP trial), (2) a different patient population (CRT non-responders in the MORE-CRT MPP trial and both CRT responders and non-responders in the MPP trial), a different endpoint (combination of HF hospitalizations and all- cause mortality in the MORE-CRT MPP trial and clinical composite score in the MPP trial), and finally the fact that the MPP trial involved acute Doppler echocardiographic measurements of the velocity-time integral of the transmitral flow, performed to verify that MPP was safe, and only patients with improved or equal haemodynamic measurements were eligible for randomization, thus potentially selecting, in both BIVP and MPP arms, long-term clinical responders and making more difficult to show differences between MPP and BIVP. As for the comparison with previous analyses of the MORE-CRT MPP trial^18^ our hypothesis is based on the fact that HF is a progressive disease, with patient conditions worsening with each new hospitalization for acute decompensation, moreover LV dysfunction is a complex condition, not limited to LV dimensions, and finally CRT benefit is based on several electrophysiological mechanisms,^28^ and not all of them directly influence LVESV. Therefore, CRT success could be more accurately measured with survival, prevention of decompensation events and disease stabilization rather than with a one dimensional dichotomous approach such as LV reverse remodelling.^29^ The limitations of the traditional approach to assessing ‘response’ to CRT on the basis of the extent of LV reverse remodelling were clearly outlined by the joint position statement published by Mullens et al.^30^ where in order to appropriately detect and report the patient trajectories after CRT implant it was advised to abandon the term ‘non-response’, by replacing it with the concept of disease modification. Furthermore, a previous secondary analysis of MORE-CRT MPP data^31^ showed that in patients with optimal pacing conditions (i.e. BIVP percentage higher than 97%) MPP was indeed associated with improved LV reverse remodelling over BIVP. This finding suggests that the assessment of CRT response in MORE-CRT MPP data,^18^ initially performed via echocardiographic measurements, could have been underpowered to show MPP value due to some patients not being treated with BIVP enough of the time, possibly due to atrial tachyarrhythmias or other conditions. This hypothesis seems to be confirmed by our results which show that the improved clinical prognosis with MPP, compared with BIVP, was higherin patients without AT/AF and in patients with a pacing percentage >97%, as shown in *Figure 4*.

### Mechanism of MPP action

Multipoint pacing has been proposed to improve the CRT response based on several possible electrophysiological mechanisms. In particular, by pacing specific LV zones and/or capturing a larger LV area, the MPP algorithm may be beneficial according to three hypotheses: (1) reducing intraventricular dyssynchrony, promoting a more coordinated LV contraction, and reducing the total time required for LV activation, (2) improving the timing of left and right ventricular contraction relative to each other and relative to atrial contraction, and therefore optimizing LV filling, (3) avoiding ischaemic scar and therefore bypassing regions with slow or blocked electrical conduction. Evidence about MPP capability to reduce intraventricular dyssynchrony (hypothesis #1) has been provided by Calò et al.^31^ who showed that MPP improves LV reverse remodelling, compared with BIVP, in patients with LV intraventricular dyssynchrony, measured as large dispersion in interventricular electrical delays along the LV lead axis. Our data, showing that the risk of HF hospitalizations and all-cause mortality is reduced with MPP in patients with long interventricular delay and in ischaemic patients (*Figure 4*), suggest that MPP might indeed improve LV-RV synchronous contraction (hypothesis #2) and improve CRT responses in patients with regions of slow or blocked electrical depolarizations due to ischaemic scars (hypothesis #3).

Previous studies have proposed interventricular electrical delay as a predictor of the CRT response.^23–25^ In particular, Gold et al.^23^ evaluated CRT responses and interventricular electrical delay in about 400 patients and showed improved LV reverse remodelling as a function of interventricular electrical delay quartiles. Our findings add new evidence to the fact that a long interventricular electrical delay, beyond being a strong predictor of de-novo CRT response,^23–25^ is also associated with improved clinical outcomes using MPP in patients who do not respond to BIVP.

### Clinical implications

Cardiac resynchronization therapy non-responders are at a higher risk of death and HF hospitalizations and cause high healthcare expenditures, compared with CRT responders.^12,20,21^ Varma et al^12^ have shown that CRT non-responders in real-world clinical practice may receive only conservative treatments which have little impact on outcomes, and rarely are indicated to specialty care or device optimization, and consequently in these patients high hospitalization rates persist. Our findings emphasize the importance of programming MPP when patients do not respond to BIVP.

Identification of patients with long interventricular delay may be important initially to screen patients who respond to CRT and to guide optimal LV pacing site but then also for programming optimization through MPP.

### Study limitations

The results of our analyses of secondary endpoints of the MORE-CRT MPP study should be interpreted as hypotheses generating. Anyhow, the large sample size and the solid statistical methods, comprising the randomized controlled design, the intention-to-treat analysis approach, and the use of multivariable methods, provide strength to our findings. HF hospitalizations and all-cause mortality were pre-specified endpoints of the study. It could seem unusual to evaluate CRT response in terms of clinical outcomes in patients who were initially classified as CRT non-responders based on echocardiographic measurements, but, on one hand, the study design required survival at 6 months and randomization of patients classified as non-responders on the basis of failed reverse remodelling, and, on the other hand, we wanted to evaluate CRT response on hard endpoints and beyond reverse remodelling.

### Study sponsor

The study was funded by Abbott Medical.

## Conclusions

Data from the MORE-CRT MPP randomized trial show that MPP is associated with clinically relevant and statistically significant reduction of HF hospitalizations or all-cause mortality in patients who do not respond to BIVP. A significantly better outcome was observed in MPP patients, compared with BIVP patients, when considering the whole study population and also in many patient subgroups, such as ischaemic patients, patients with very wide (≥160 ms) QRS, patients with long (>105 ms) interventricular electrical delay, and patients without atrial tachyarrhythmias.

## Supplementary Material

euaf070_Supplementary_Data

## Data Availability

The data underlying this article were provided by Abbott. Data will be shared on reasonable request to the corresponding author with permission of Abbott.
