# Supplementary material for: Multipoint pacing is associated with reduction of heart failure hospitalizations or death in patients who do not respond to cardiac resynchronization therapy: results of the MORE-CRT MPP randomized trial
Source: Europace. 2025 Mar 30;27(6):euaf070. doi: 10.1093/europace/euaf070 (PMC12131796; doi:10.1093/europace/euaf070)
Supplement: euaf070_Supplementary_Data [file euaf070_supplementary_data.docx]

***Supplementary Data***

Inclusion criteria

1. Met ESC Guidelines or ACCF/AHA/HRS Class I or Class IIa indications for CRT implant (including upgrades from single or dual chamber ICDs)
2. Willing and able to comply with study requirements
3. Indicated their understanding of the study and willingness to participate by signing an appropriate informed consent form

Exclusion Criteria

1. Previous CRT device implanted
2. Myocardial Infarction, unstable angina within 40 days prior the enrollment
3. Recent cardiac revascularization (PTCA, Stent or CABG) in the 4 weeks prior to enrollment or planned for the 3 months following implant
4. Cerebrovascular accident or transient ischemic attack in the 3

months prior the enrollment

1. Primary valvular disease requiring surgical correction
2. Persistent atrial fibrillation
3. Permanent atrial fibrillation not treated with AV node ablation within 2 weeks from implant
4. History of paroxysmal atrial fibrillation within 30 days prior the enrollment
5. Unable to comply with the follow-up schedule
6. Less than 18 years of age
7. Pregnant or are planning to become pregnant during the duration of the investigation
8. Classification of Status 1 for cardiac transplantation or consideration for

transplantation over the 12 months following enrollment

1. Undergone a cardiac transplantation
2. Life expectancy < 12 months Participating in any other clinical investigation

**Table A – Patients characteristics (showing p values for the comparison of MPP and BIVP)**

|  | **Subjects Analyzed (N=1421)** | **MPP ON (N=722)** | **MPP OFF (N=699)** | **p value** |
| --- | --- | --- | --- | --- |
|  |  |  |  |  |
| **Age in years, mean ± st dev (N)** | 68 ± 10 (1421) | 68 ± 10 (722) | 68 ± 11 (699) | 0.8850 |
| **Female Gender, % (n/N)** | 23.1 (328/1421) | 21.7 (157/722) | 24.5 (171/699) | 0.2241 |
| **NYHA Class, % (n/N)** | | | | 0.5962 |
| **NYHA Class II, % (n/N)** | 49.0 (697/1421) | 50.1 (362/722) | 47.9 (335/699) |  |
| **NYHA Class II, % (n/N)** | 48.5 (689/1421) | 47.2 (341/722) | 49.8 (348/699) |  |
| **NYHA Class II, % (n/N)** | 2.2 (31/1421) | 2.4 (17/722) | 2.0 (14/699) |  |
| **Ischemic cardiomyopathy, % (n/N)** | 51.0 (725/1421) | 52.5 (379/722) | 49.5 (346/699) | 0.2591 |
| **Hypertension, % (n/N)** | 61.7 (877/1421) | 61.9 (447/722) | 61.5 (430/699) | 0.8783 |
| **Hypercholesterolemia, % (n/N)** | 42.7 (607/1421) | 45.2 (326/722) | 40.2 (281/699) | 0.0592 |
| **Diabetes Mellitus, % (n/N)** | 37.7 (536/1421) | 36.8 (266/722) | 38.6 (270/699) | 0.4878 |
| **COPD, % (n/N)** | 10.7 (152/1421) | 10.5 (76/722) | 10.9 (76/699) | 0.8327 |
| **Renal Disease, % (n/N)** | 19.4 (276/1421) | 19.9 (144/722) | 18.9 (132/699) | 0.6134 |
| **Active Smoker, % (n/N)** | 9.7 (138/1421) | 9.7 (70/722) | 9.7 (68/699) | 0.9833 |
| **Ex-Smoker, % (n/N)** | 35.3 (501/1421) | 34.2 (247/722) | 36.3 (254/699) | 0.4015 |
| **Stroke, % (n/N)** | 4.2 (59/1421) | 5.0 (36/722) | 3.3 (23/699) | 0.1092 |
| **Transient Ischemic Attack, % (n/N)** | 3.2 (46/1421) | 4.2 (30/722) | 2.3 (16/699) | 0.0469 |
| **Peripheral Artery Disease, % (n/N)** | 5.8 (83/1421) | 5.4 (39/722) | 6.3 (44/699) | 0.4730 |
| **Neoplastic Disease, % (n/N)** | 4.0 (57/1421) | 4.0 (29/722) | 4.0 (28/699) | 0.9916 |
| **Thyroid Dysfunction, % (n/N)** | 9.5 (135/1421) | 9.1 (66/722) | 9.9 (69/699) | 0.6390 |
| **LBBB, % (n/N)** | 63.2 (781/1236) | 62.8 (391/623) | 63.6 (390/613) | 0.7538 |
| **RBBB, % (n/N)** | 7.7 (95/1236) | 8.8 (55/623) | 6.5 (40/613) | 0.1286 |
| **Left Anterior Fascicular Block, % (n/N)** | 6.7 (83/1236) | 6.3 (39/623) | 7.2 (44/613) | 0.5192 |
| **Left Posterior Fascicular Block, % (n/N)** | 0.2 (2/1236) | 0.3 (2/623) | 0.0 (0/613) | 0.4996 |
| **Intra Ventricular Conduction Delay, % (n/N)** | 24.1 (298/1236) | 23.8 (148/623) | 24.5 (150/613) | 0.7693 |
| **QRS duration in ms, mean ± st dev (N)** | 155 ± 25 (1421) | 155 ± 26 (722) | 155 ± 24 (699) | 0.8804 |
| **LVESV (ml) mean ± st dev (N)** | 155 ± 66 (1389) | 155 ± 65 (710) | 155 ± 67 (679) | 0.8562 |
| **LVEDV (ml) mean ± st dev (N)** | 216 ± 77 (1389) | 216 ± 77 (710) | 215 ± 78 (679) | 0.8086 |
| **LVEF %, mean ± st dev (N)** | 29 ± 8 (1389) | 29 ± 8 (710) | 29 ± 8 (679) | 0.4743 |
| **CRT-D, % (n/N)** | 91.8 (1305/1421) | 91.7 (662/722) | 92.0 (643/699) | 0.8371 |

**Table B – Pharmacological therapy**

|  | **Subjects Analyzed (N=1421)** | **MPP ON (N=722)** | **MPP OFF (N=699)** | **p value** |
| --- | --- | --- | --- | --- |
|  |  |  |  |  |
| **Medication, % (n/N)** | | | |  |
| ACE | 59.5% (846/1421) | 60.2% (435/722) | 58.8% (411/699) | 0.5774 |
| Aldosterone Antagonist | 37.9% (538/1421) | 38.9% (281/722) | 36.8% (257/699) | 0.4029 |
| ARBs | 32.2% (458/1421) | 32.3% (233/722) | 32.2% (225/699) | 0.9734 |
| Antiarrhythmics | 17.6% (250/1421) | 16.2% (117/722) | 19.0% (133/699) | 0.1625 |
| Anticoagulants | 28.8% (409/1421) | 28.4% (205/722) | 29.2% (204/699) | 0.7419 |
| Antiplatelets | 53.3% (757/1421) | 55.3% (399/722) | 51.2% (358/699) | 0.1263 |
| Beta-Blockers | 87.9% (1249/1421) | 88.1% (636/722) | 87.7% (613/699) | 0.8209 |
| Calcium Channel Blockers | 6.4% (91/1421) | 6.2% (45/722) | 6.6% (46/699) | 0.7887 |
| Cardiac Glycosides | 5.4% (77/1421) | 5.1% (37/722) | 5.7% (40/699) | 0.6187 |
| Diuretics | 80.1% (1138/1421) | 80.3% (580/722) | 79.8% (558/699) | 0.8120 |
| Nitrates | 8.3% (118/1421) | 7.8% (56/722) | 8.9% (62/699) | 0.4469 |
| Statins | 61.4% (873/1421) | 62.7% (453/722) | 60.1% (420/699) | 0.3037 |

**Figure A – Raw incidence of clinical events in the 1677 randomized patients**

**
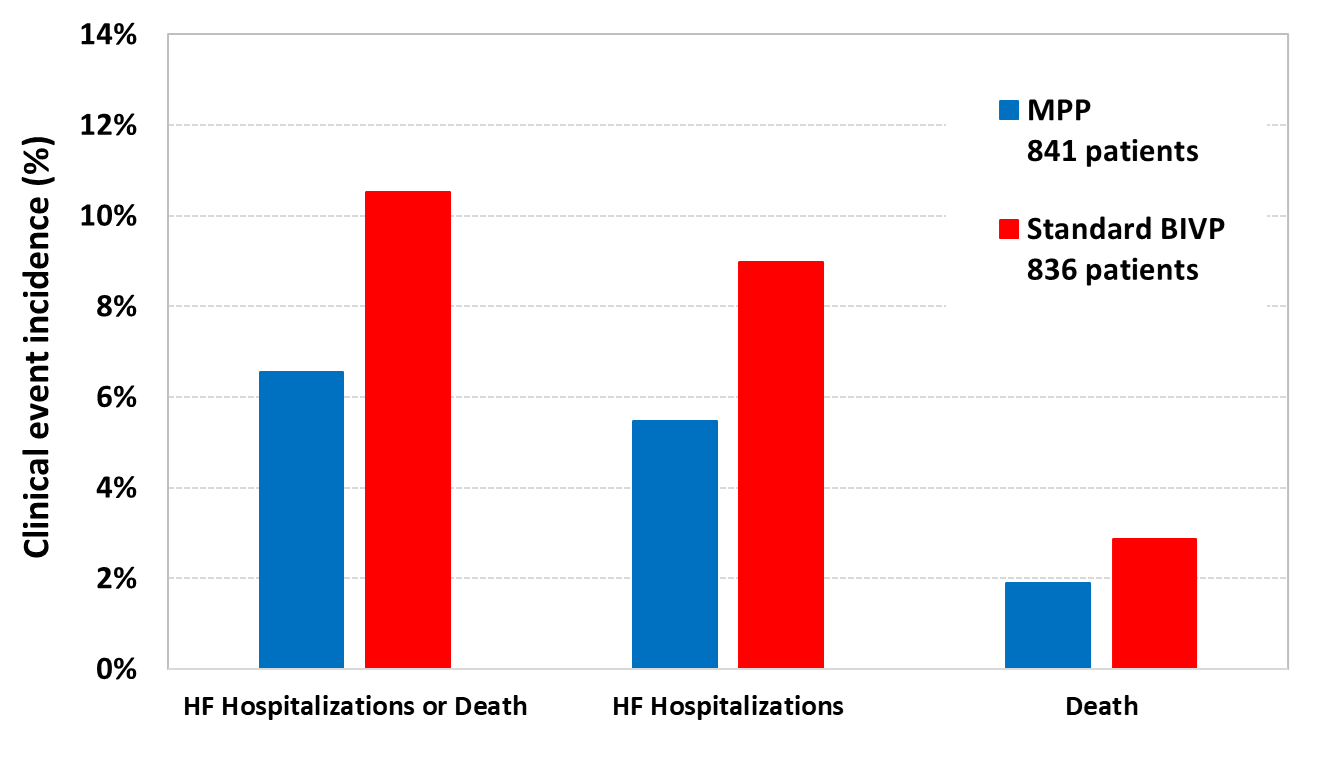
**

**p = 0.0035**

**p = 0.0056**

***List of participating institutions and principal investigators in the MORE-CRT MPP trial***

| **Investigational Site** | **Principal Investigator** |
| --- | --- |
| Royal Adelaide Hospital | Sanders, Prash |
| The Alfred Hospital | Mariani, Justin |
| St. Andrews War Memorial Hospital | Hayes, John |
| Westmead Hospital | Sivagangabalan, Gopal |
| Klinik Floridsdorf | Achleitner, Reinhard |
| Wilhelminenspital Wien | Koch, Johannes |
| Krankenhaus der Stadt St. Polten | Thudt, Karin |
| Hopital Erasme | Casado, Ruben |
| St. Joseph Gilly | Leroy, Jean |
| Institut de Cardiologie de Montreal (Montreal Heart Inst.) | Thibault, Bernard |
| Rouge Valley Centenary | Janmohamed, Amir |
| St. Paul's Hospital | Chakrabarti, Shanta |
| Institut de Cardiologie de Quebec (Hopital Laval) | Molin, Franck |
| HSC, Eastern Health | Connors, Sean |
| QE II Health Sciences | Sapp, John |
| Kingston General Hospital | Simpson, Christopher |
| CHUM | Coutu, Benoit |
| McGill University Health Centre General Hospital | Essebag, Vidal |
| Royal Alexandra Hospital | Williams, Randall |
| St. Michael's Hospital | Mangat, Iqwal |
| Foothills Medical Centre | Sumner, Glen |
| CHUS Fleurimont | Ayala-Paredes, Felix |
| Prince of Wales Hospital | Chan, Joseph Yat Sun |
| Angiografia Clinica de Occidente | Dager Gomez, Antonio |
| Aalborg Sygehus Syd | Sogaard, Peter |
| Odense University Hospital | Johansen, Jens Brock |
| Skejby University Hospital | Nielsen, Jens |
| Turku University Hospital | Lund, Juha |
| Keski-Suomi Central Hospital | Nyman, Kai |
| CHRU Hopital de Pontchaillou | Leclercq, Christophe |
| CHRU Albert Michallon | Defaye, Pascal |
| CHRU Lille | Marquie, Christelle |
| Centre Cardiologique Du Nord | Piot, Olivier |
| Medipole Lyon-Villeurbanne | Poty, Herve |
| CHRU de Brest | Mansourati, Jacques |
| Hopital Clairval | Mechulan, Alexis |
| Hopital Pitie Salpetriere | Hidden-Lucet, Franciose |
| Hopital Prive du Confluent | Gras, Daniel |
| Hopital Saint Philibert | Guyomar, Yves |
| CHU - Montpellier | Pasquie, Jean-Luc |
| CHRU Rouen Hospital Charles Nicolle | Anselme, Frederic |
| CHU de Nancy - Hopital de Brabois | Blangy, Hugues |
| CHU Hopital G. & R. Laennec | Lande, Gilles |
| CHR de La Reunion - Site du CHFG | Clerici, Gael |
| Centre Hospitalier de Belfort-Montbeliard | Fouche, Renaud |
| Institute Cardio. Paris-Sud - Institut Jacques Cartier | Horvilleur, Jerome |
| CHRU Hopital de la Cote de Nacre | Pellissier, Arnaud |
| Hopital Saint Joseph | Gitenay, Edouard |
| CHU Rangueil Toulouse | Mondoly, Pierre |
| Centre Hospitalier de Valence | Miralles, Aurelien |
| CHU du Bocage | Laurent, Gabriel |
| CHU Gabriel Montpied | Eschalier, Romain |
| CHU Trousseau | Babuty, Dominique |
| Deutsches Herzzentrum Munchen des Freistaates Bayern | Kolb, Christof |
| Medizinische Hochschule Hannover | Veltmann, Christian |
| Medizinische Einrichtungen der Universitat zu Koln | Steven, Daniel |
| Berufsgenossenschaftliche Kliniken Bergmannsheil | Boesche, Leif |
| Herz-und Diabetes Zentrum NRW | Sommer, Philipp |
| Universitatsklinikum Leipzig AOR | Neef, Martin |
| Universitatsmedizin Berlin - Campus Benjamin Franklin (CBF) | Huemer, Martin |
| Evangelisches Krankenhaus Kalk gGmbH | Eberhardt, Frank |
| Schuchtermann-Schiller'sche Kliniken GmbH & Co. KG | Moennig, Gerold |
| Universitatsklinikum Munster | Eckardt, Lars |
| Klinikum Bielefeld gGmbH Klinikum-Mitte | Stellbrink, Christoph |
| Markische Kliniken GmbH Klinikum Ludenscheid | Lemke, Bernd |
| Klinikum Ingolstadt GmbH | Seidl, Karlheinz |
| Helios-Klinikum Erfurt GmbH | Schade, Anja |
| Klinikum Coburg GmbH | Brachmann, Johannes |
| Krankenhaus der Barmherzigen Bruder | Voss, Frederik |
| Universitatsklinikum Greifswald | Busch, Mathias |
| Herz- und Gefäßzentrum am Krankenhaus Neu-Bethlehem | Hansen, Claudius |
| Kerckhoff-Klinik gGmbH | Sperzel, Johannes |
| St.-Marien-Hospital GmbH | Perings, Christian |
| Kliniken der Friedrich-Alexander-Universitat | Arnold, Martin |
| Charite Campus Virchow Klinikum | Blaschke, Florian |
| Hegau-Bodensee-Hochrhein-Kliniken GmbH | Kollum, Marc |
| Albertinen-Krankenhaus Hamburg | Naegele, Herbert |
| Stadtisches Klinikum Gutersloh gGmbH | Er, Fikret |
| St. Vinzenz-Hospital | Winter, Stefan |
| Universitatsklinikum Wurzburg | Nordbeck, Peter |
| Universitatsmedizin Gottingen Georg-August-Universitat | Herting, Jonas |
| Kliniken Villingen-Schwenningen | Jung, Werner |
| Klinikum St. Georg gGMbH | Klein, Norbert |
| Elisabeth-Krankenhaus Essen GmbH | Schmitz, Dietmar |
| Klinikum Oldenburg gGmbH | Oswald, Hanno |
| Universitats-Herzzentrum Freiburg - Bad Krozingen | Restle, Christian |
| The Onassis Cardiac Center | Theodorakis, George |
| Escorts Heart Institute & Research Centre | Saxena, Anil |
| CARE Banjara | Narasimhan, Calambur |
| Medanta - The Medicity Hospital | Bhargava, Kartikeya |
| Care Institute of Medical Sciences | Naik, Ajay |
| Apollo Hospital | Karthigesan, Arumugam Murugesan |
| Pushpawati Singhania Hospital & Research Institute | Kler, Tarlochan |
| Medanta Medicity Hospital | Singh, Balbir |
| Fortis Hospital | Pal, Shashidhar |
| Postgraduate Institute of Medical Education & Research | Vijayvergiya, Rajesh |
| Asian Institute of Gastroenterology (AIG) Hospital | Narasimhan, Calambur |
| Sheba Medical Center | Bar Lev, David |
| Rabin Medical Center | Golovchiner, Gregory |
| Tel Aviv Medical Center | Viskin, Samuel |
| Hadassah - Ein Kerem | Luria, David |
| Casa di Cura Dpott. Pederzoli | Vicentini, Alfredo |
| Policlinico Casilino | Calo, Leonardo |
| Azienda Ospedaliero Universitaria Pisana | Bongiorni, Maria Grazia |
| Ospedale Giovanni Paolo II - Cardiology | Nicosia, Antonino |
| Ospedale Civile Maggiore di Verona Borgo Trento | Morani, Giovanni |
| Ospedale dei Pellegrini | Ducceschi, Valentino |
| Azienda Ospedaliera Di Venere | Bonfantino, Vincenzo |
| Az Osp.Universitaria Maggiore della Carita | Dell'era, Gabriele |
| Ospedale S. Giovanni Bosco | Capogrosso, Paolo |
| Ospedale di Portogruaro | Nangah, Rene |
| Presidio Osp. Vito Fazzi | Pisano, Ennio |
| Universita degli Studi di Padova | Bertaglia, Emanuele |
| Casa Sollievo della Sofferenza | Potenza, Domenico Rosario |
| Az. Osp. Spedali Civili di Brescia | Curnis, Antonio |
| Az.Osp.Universitaria Consorziale Policlinico | Favale, Stefano |
| Clinica Mediterranea | Iuliano, Assunta |
| Azienda Ospedaliera S.Anna e S.Sebastiano | Viscusi, Miguel |
| Policlinico S.Orsola Malpighi | Ziacchi, Matteo |
| Az. Osp.Sant'Anna | Russo, Giovanni |
| Ospedale Madre G. Vannini | Ansalone, Gerardo |
| Ospedale S. Giovanni Calibita Fatebenefratelli di Roma | Bianchi, Stefano |
| St. Marianna University School of Medicine Hospital | Harada, Tomoo |
| American University of Beirut Medical Center | Khoury, Maurice |
| Institut Jantung Negara | Razali, Omar |
| Mater Dei Hospital | Aquilina, Oscar |
| Isala - ZWolle | Delnoy, Peter-Paul |
| Amsterdam Academic Medical Centre (AMC) | Knops, Reinoud |
| Catharina Ziekenhuis | Houthuizen, Patrick |
| TweeSteden Ziekenhuis - Locatie Tilburg | Widdershoven, J.W.M.G. |
| Medisch Spectrum Twente | Van Es, Jan |
| Gornoslaskie Centrum Medyczne im.prof. Leszka Gieca | Wilczek, Jacek |
| Wojewodzki Specjalistyczny Szpital im. Bieganskiego | Bednarkiewicz, Zbigniew |
| Slaskie Centrum Chorob Serca | Kalarus, Zbigniew |
| Szpital Kliniczny Przemienienia Panskiego UM w Poznaniu | Mitkowski, Przemyslaw |
| Santa Maria Hospital | Marques, Pedro |
| Centro Hospitalar Vila Nova Gaia | Primo, Joao |
| Hospital de Braga | Rocha, Sergia |
| Hospital Garcia de Orta, EPE | Brand?o, Luis |
| Hospital de Santa Cruz | Adragao, Pedro |
| Centro Hospitalar do Alto Ave, Unidade de Guimarães | Sanfins, Victor |
| Heart Rhythm Management | Sotomonte Ariza, Juan |
| Hospital San Lucas Ponce | Perez, Francisco |
| Meshalkin National Medical Research Center | Romanov, Alexander |
| King Fahad Medical City | Al Samadi, Faisal |
| King Fahad Armed Forces Hospital | Bokhari, Fayez |
| National University Hospital | Seow, Swee Chong |
| Yonsei University Health System | Joung, Bo Young |
| Samsung Medical Center | Park, Seung-Jung |
| Sejong Hospital | Park, Mi Young |
| Seoul National University Bundang Hospital | Oh, Il Young |
| Seoul National University Hospital | Oh, Seil |
| Seoul St. Mary's Hospital | Oh, Yong-Seog |
| Hospital Universitario Infanta Cristina | Fernandez Concha, Joaquin |
| Hospital Universitari i Politecnic La Fe | Osca Asensi, Joaquin |
| Fundacion Jimenez Diaz | Sanchez Borque, Pepa |
| HCU Virgen de la Victoria | Alzueta Rodriguez, Javier |
| Hospital Ramon y Cajal | Hernandez Madrid, Antonio |
| Hospital Alvaro Cunqueiro, Dept of EP & Arrhythmias | Garcia Campo, Enrique |
| Hospital General Universitario Gregorio Marañon | Arenal, Angel |
| Hospital Universitario Puerta de Hierro | Toquero-Ramos, Jorge |
| Hospital de la Santa Creu I Sant Pau | Vinolas, Xavier |
| Hospital Universitario Doce de Octubre | Lopez-Gil, Maria |
| Hospital Universitario Virgen de la Nieves | Macias, Maria Rosa |
| Hospital Universitario A Coruña | Mosquera, Ignacio |
| Complexo Hospitalario Universitario de Santiago | Martinez Sande, Jose |
| Hospital Universitario Miguel Servet | Oloriz, Teresa |
| Karolinska University Hospital, Solna | Gadler, F |
| Hopital Cantonal Universitaire de Geneva | Burri, Haran |
| National Taiwan University Hospital | Ho, Li Ting |
| Chang Gung Memorial Hospital | Wang, Chun-Chieh |
| John Radcliffe Hospital | Betts, Tim |
| Golden Jubilee National Hospital | Gardner, Roy |
| The Royal Sussex County Hospital | Ellery, Sue Mary |
| Southampton University Hospital | Flett, Andrew |
| Manchester Heart Center, Manchester Royal Infirmary | Muhyaldeen, Sahrkaw |
| The Great Western Hospital | Foley, Paul |
| Queen Elizabeth Hospital | Leyva-Leon, Francisco |
| St. Thomas Hospital | Rinaldi, Christopher |
| Kings College Hospital | Scott, Paul |
| Wansbeck General Hospital | Runnett, Craig |
| Cardiac Rhythm Specialists, Inc. | Polosajian, Leo |
| Heart Center Research, LLC. | Jennings, John |
| Lancaster General Hospital | Bansal, Sandeep |
| Baptist Health Lexington | Tomassoni, Gery |
| Redmond Regional Medical Center | Styperek, Robert |
| Cardiovascular Associates of Mesa | Kaplan, Andrew |
| Colorado Heart & Vascular, P.C. | Venkataraman, Ganesh |
| Samaritan Heart & Vascular Institute - Cardiology Dept. | Hsing, Jeff |
| Glendale Adventist Medical Center | Mckenzie, John |
| St. Francis Hospital | Sellers, Matthew |
| Comprehensive Cardiovascular | Habib, Moksedul |
| EP Heart | Hariharan, Ramesh |
| Erlanger Medical Center | Manyam, Harish |
| Phoenix Cardiovascular Research Group | Bahu, Marwan |
| Baylor All Saints Medical Center at Fort Worth | Shah, Syed |
| Central Cardiology | Salvo, Jared |
| McLaren Health Care Corporation | Buerkel, Daniel |
| Memorial Katy Cardiology Associates | Kashani, Amir |
| Deborah Heart & Lung Center | Corbisiero, Raffaele |
| Scripps Health | Rogers, John |
| Vivek Mangla, MD | Mangla, Vivek |
| Coliseum Medical Centers | Hoffman, Jonathan |
| MidMichigan Medical Center-Midland | Islam, Nilofar |
| San Diego Cardiac Center | Athill, Charles |
| Brigham & Women's Hospital | Koplan, Bruce |
| Cardiology Associates of Fairfield County, PC | Tiano, Joseph |
| Cardiovascular Associates of the Delaware Valley | Levi, Steven |
| Eisenhower Medical Center | Feldman, Leon |
| Methodist University Hospital | Jha, Sunil |
| Sansum Clinic - Santa Barbara Medical Foundation | Cogert, Gregory |
| St. Elizabeth Medical Center - South Unit | Sinno, Mohamad |
| Munson Medical Center | Jaffe, Brian |
| CHI Health Creighton University Medical Center-Bergan Mercy | Abuissa, Hussam |
| St. Vincent Heart Clinic Arkansas | Chakka, Mangaraju |
